# Measurement of the Inner Macular Layers for Monitoring of Glaucoma: Confounding Effects of Age-Related Macular Degeneration

**DOI:** 10.1016/j.ogla.2022.06.006

**Published:** 2022-06-21

**Authors:** Leila Chew, Vahid Mohammadzadeh, Massood Mohammadi, Veronica Toriz, Nancy Rosa, Michael B. Gorin, Navid Amini, Kouros Nouri-Mahdavi

**Affiliations:** 1 Glaucoma Division, Stein Eye Institute, David Geffen School of Medicine, University of California Los Angeles, Los Angeles, California; 2 Department of Computer Science, California State University Los Angeles, Los Angeles, California; 3 Retinal Disorders and Ophthalmic Genetics Division, Stein Eye Institute, David Geffen School of Medicine, University of California Los Angeles, Los Angeles, California

**Keywords:** Age-related macular degeneration, Drusen, Glaucoma, Inner nuclear layer, Inner retinal layers, Inner plexiform layer, Macula, OCT, Optical coherence tomography, Retinal atrophy

## Abstract

**Objective:**

To investigate the confounding effect of nonexudative age-related macular degeneration (AMD), specifically drusen and outer retinal atrophy, on the architecture and automated segmentation of the inner retinal layers as measured with OCT.

**Design:**

Observational cross-sectional study.

**Subjects:**

Two hundred sixty-three consecutive eyes with nonexudative AMD were identified through a retrospective chart review. Exclusion criteria were a diagnosis of glaucoma or glaucoma suspect, other retinal pathology affecting the macula, axial length > 26.5 mm or spherical equivalent less than −6 diopters, any other optic nerve or neurologic disorders, or poor image quality.

**Methods:**

Drusen were automatically segmented on macular OCT B-scans with a publicly available and validated deep learning approach. Automated segmentation of the inner plexiform layer (IPL)/inner nuclear layer (INL) boundary was carried out with the device’s proprietary software.

**Main Outcome Measures:**

Quality of segmentation of the IPL/INL boundary as a function of drusen size and presence of inner retinal layer displacement in the area of macular pathology (drusen or atrophy).

**Results:**

One hundred twenty-five eyes (65 patients) met the inclusion criteria. Drusen size varied between 16 and 272 μm (mean, 118 μm). Automated segmentation had a 22% chance of failure if the drusen height was between 145 and 185 μm and was most likely to fail with drusen heights above 185 μm. When drusen height was normalized by total retinal thickness, segmentation failed 36% of the time when the drusen to total retinal thickness ratio was 0.45 or above. Images were likely to show displacement of inner retinal layers with drusen heights above 176 μm and a normalized drusen height ratio of 0.5 or higher. Eighty-seven percent of images with outer retinal atrophy displayed incorrect segmentation.

**Conclusions:**

Outer retinal diseases can alter the retinal topography and affect the segmentation accuracy of the inner retinal layers. Large drusen may cause segmentation error and compression of the inner macular layers. Geographic atrophy confounds automated segmentation in a high proportion of eyes. Clinicians should be cognizant of the effects of outer retinal disease on the inner retinal layer measurements when interpreting the results of macular OCT imaging in patients with glaucoma.

OCT provides high-resolution images of the retina and plays a crucial role in monitoring ophthalmic diseases. Macular OCT is the standard modality for the evaluation of central retinal ganglion cells.^1^ There is increasing evidence to support the use of macular OCT for monitoring glaucoma.^1–4^ Different macular layers have been evaluated in various studies to date. These include full macular thickness, ganglion cell complex (GCC), ganglion cell–inner plexiform layer (GCIPL), and the ganglion cell layer proper. The GCC is comprised of the macular retinal nerve fiber layer (RNFL), ganglion cell layer, and inner plexiform layer (IPL). The GCIPL includes the ganglion cell layer and IPL only and does not include the RNFL. A meta-analysis by Kansal et al^2^ evaluated 150 studies with 16 104 glaucomatous eyes and 11 543 control eyes and found similar diagnostic accuracy for detection of glaucoma among circumpapillary RNFL, GCIPL, and GCC thickness.

The role of macular OCT imaging is especially compelling in advanced stages of glaucoma. Some macular retinal ganglion cells may persist later in the course of the disease because the circumpapillary RNFL reaches its measurement floor earlier.^2,3,5^ In advanced glaucomatous eyes, Hammel et al^6^ demonstrated higher rates of change for GCIPL measurements compared with circumpapillary RNFL. The measurement of inner macular layer thickness relies on accurate segmentation of the individual retinal layers. This is typically carried out automatically by built-in software algorithms available within the OCT devices. Several retinal pathologies, such as macular edema and epiretinal membrane, can affect the accuracy of the segmentation of these layers.^7,8^ However, it is unclear how the pathology of the outer retina affects the analysis of the inner retinal layers, and therefore, affects the interpretation of damage due to glaucoma or its progression.

Age-related macular degeneration (AMD) is one of the most frequent diseases affecting the outer retina and is a leading cause of vision loss in older adults.^9,10^ It is a degenerative disease that mainly affects the macula and is characterized by the presence of drusen, extracellular deposits derived from immune-mediated and metabolic processes, retinal pigment epithelium (RPE) changes and atrophy, and extensive areas of geographic atrophy in advanced stages.^9^ Drusen and atrophy of the outer layers of the macula can alter the architecture of the macula and may interfere with automatic segmentation of the inner retinal layers.^11,12^ Outer retinal deposits may cause the compression of the inner retinal layers, and hence, thickness measurements of the inner retina may not reflect true neural tissue content.^13,14^

The purpose of this study was to investigate the effect of nonexudative AMD, specifically drusen and outer retinal atrophy, on the architecture and segmentation of the inner retinal layers in eyes without any pathology affecting the inner retina.

## Methods

We carried out a retrospective chart review of patients seen over a 1-year period (May 26, 2020, to May 26, 2021) by a single retina specialist (M.B.G.) at the Retinal Disorders and Ophthalmic Genetics Division of the Stein Eye Institute at UCLA. This study was approved by UCLA’s institutional review board. Waiver of informed consent was granted for the purposes of data collection. The described research adhered to the tenets of the Declaration of Helsinki.

All patients had a diagnosis of nonexudative AMD based on the retina clinician’s records. Exclusion criteria were: (1) diagnosis of glaucoma, glaucoma suspect, or ocular hypertension; (2) other retinal pathology affecting the macula; (3) high myopia, defined as axial length > 26.5 mm or spherical equivalent less than −6 diopters; (4) any other optic nerve or neurological disorders; (5) poor image quality, defined as quality score < 25 dB on Spectralis OCT images. Two hundred sixty-three eyes with nonexudative AMD were initially identified. Among them, 138 eyes were excluded after applying the exclusion criteria, as described in Figure S1 (available at www.ophthalmologyglaucoma.org).

Eyes were classified as early, intermediate, or advanced AMD based on the International Classification of Diseases (ICD)-10 diagnostic codes. There were 14 eyes that were not classified with an ICD-10 code at the time of the encounter. For these eyes, ICD-10 code criteria were applied retrospectively using clinical examination notes and the available images. Early-stage AMD was defined as multiple small drusen, few intermediate drusen, or RPE abnormalities. Intermediate stage AMD was defined as the presence of extensive intermediate drusen or at least one large drusen. Advanced atrophic stage AMD was defined as the presence of geographic atrophy, separated into groups with and without subfoveal involvement.

A reviewer (L.C.) classified eligible eyes as having predominantly drusen or predominantly atrophic areas on OCT images, and 3 reviewers (K.N.M., V.M., M.M.) subsequently reviewed borderline images to adjudicate the classification. There were 11 images with concomitant atrophy and drusen. These were categorized into predominantly drusen (6 eyes), predominantly atrophy (1 eye), distinct areas of atrophy and drusen that could be analyzed separately in both groups (2 eyes), and overlying atrophy and drusen leading to exclusion to avoid confounding of analysis (2 eyes).

A total of 98 eyes were classified as having drusen and 31 eyes had atrophy. All images with drusen height larger than 125 μm were included for analysis, whereas only a representative subset of images with smaller drusen (< 125 μm) was included. An initial review showed that the segmentation algorithm of the OCT device mostly failed with increasing drusen height, and most small drusen did not cause noticeable changes in the machine’s ability to delineate inner retinal layers. Thus, for the purpose of analyzing the effect of drusen on segmentation error, we decided to retain all eyes with larger drusen but only a representative group of eyes with smaller drusen. In case more than one druse was present on a single B-scan, only the effect of the tallest druse was further analyzed.

### OCT Imaging and Drusen Segmentation

OCT images were acquired with Spectralis spectral domain OCT (Heidelberg Engineering). Macular cube scans (30° × 25° in size) centered on the fovea were obtained, yielding 61 B-scans spaced approximately 120 μm apart. One single B-scan from each cube scan displaying a druse with the greatest height was selected. Each B-scan was composed of 768 A-scans. B-scans were repeated 9 to 11 times to decrease the speckle noise and improve the resolution.

Drusen were automatically segmented using a publicly available deep learning approach proposed and validated by Gorgi Zadeh et al.^15^ At first, the drusen segmentation approach automatically segmented the RPE and Bruch’s membrane (BM) layers, using a convolutional neural network, which transformed an input B-scan into RPE and BM probability maps. For the final hard segmentation of RPE and BM layers, probability maps were converted into cost maps so that pixels with higher probability have lower costs. Dijkstra’s algorithm was then used to find a path with the minimum accumulated costs from the left to the right of each map. The extracted paths were considered final RPE and BM layer segmentation. In the second step, an ideal (normal) RPE was estimated through a rectification of the RPE and BM bands. In the rectification step, both the RPE and BM were shifted vertically and column-wise, until the BM layer became a straight horizontal line, then a low-degree polynomial was fitted on the shifted RPE layer and transformed back into the original image coordinates and was regarded as the drusen-free RPE. Finally, any area between the RPE and drusen-free RPE was identified as drusen. The location and height of the druse with maximum height in a representative B-scan within each volume scan was determined and used for analysis. The drusen height values were normalized by dividing the drusen height by the total retinal thickness at the location of the drusen as defined by the shortest perpendicular distance from the outer RPE-BM complex to the inner limiting membrane.

Automatic segmentation of the inner limiting membrane and the inner plexiform layer (IPL)/inner nuclear layer (INL) boundary, the 2 layers enclosing the GCC, was conducted via Glaucoma Module Premium Edition software, a built-in software application in Spectralis OCT, which delineates individual layers of the macula. The boundaries of the GCC were examined in each image. Each image was then independently evaluated by 2 experienced clinicians to determine whether it was correctly or incorrectly segmented as well as whether there was any evidence of displacement of the inner retinal layers. Consensus was not initially reached for 4 images regarding segmentation and 12 images regarding displacement. These images were then reviewed by the 2 original reviewers and another clinician and adjudicated.

### Statistical Analyses

Parametric and nonparametric summary parameters were calculated for demographic and clinical features as indicated. Histograms and dot plots were used for visualization purposes. The unpaired *t* test was used to compare drusen height measurements across groups of eyes with and without inner retinal displacement or with or without altered segmentation.

## Results

Table 1 provides the demographic characteristics of the patients in this study. The average patient age was 78.0 ± 9.8 years. There were 31 men and 34 women. Eighty percent of the patients were White. The most frequent AMD stage as determined by ICD-10 diagnostic code criteria was the intermediate stage, followed by advanced atrophic without subfoveal involvement. The low frequency of early-stage AMD eyes is due to inclusion of eyes with mostly larger drusen.

### Effect of Drusen on Inner Macular Layers on OCT B-scans

**Figure 1.**
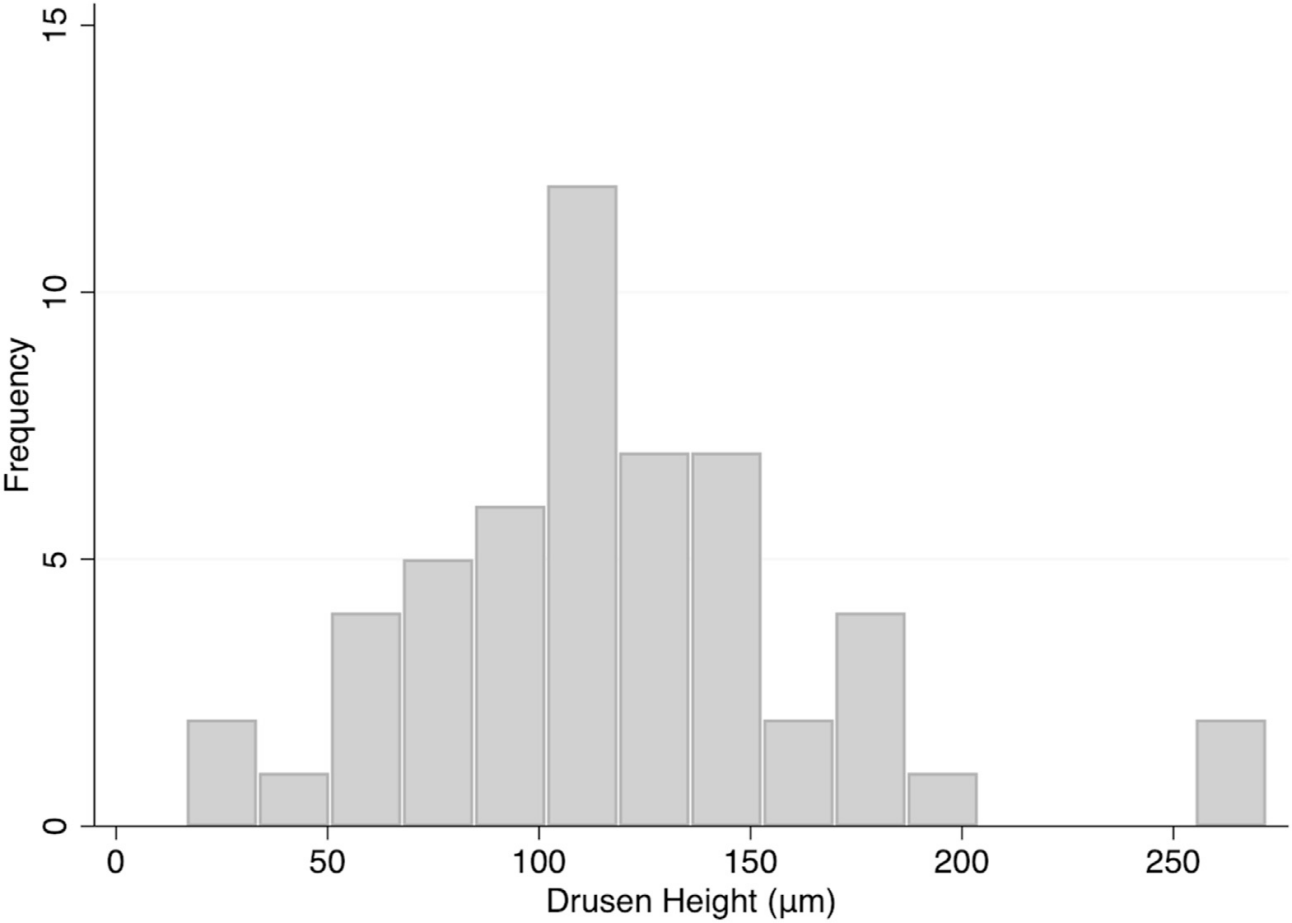
Distribution of drusen height in the subset of eyes with drusen on OCT images (53 eyes).

**Figure 2.**
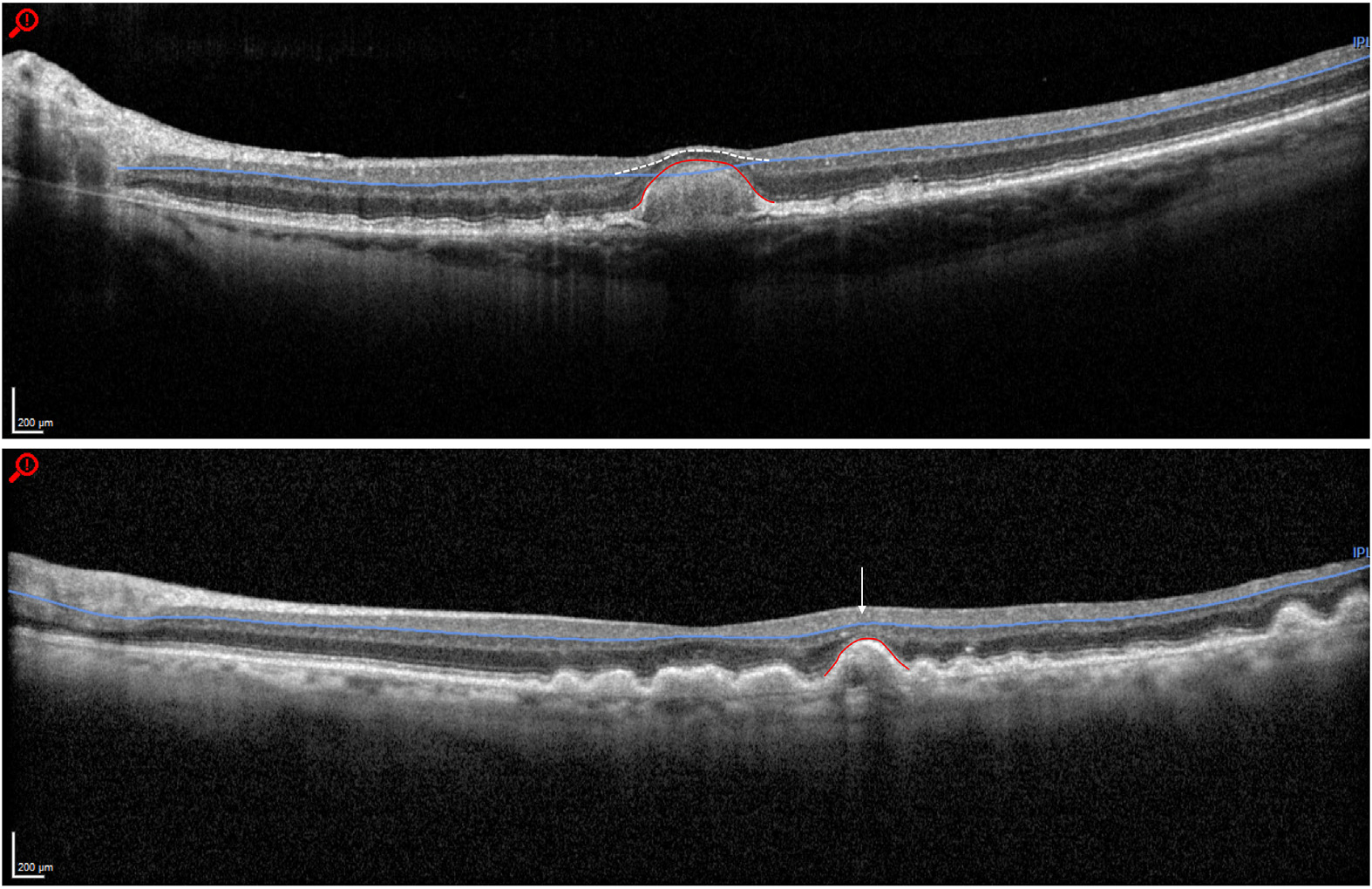
Examples of the effect of large drusen on the inner retinal layers. Top, large drusen (289 μm in height) causing compression of the inner retinal layers and incorrect segmentation of the outer boundary of the ganglion cell complex. The white dashed line demonstrates the correct inner plexiform/inner nuclear layer boundary. The red line demarcates the border of the drusen. Bottom, multiple large drusen (largest 186 μm in height) causing distortion of the tissue architecture but with correct segmentation. The arrow highlights an area of inner retinal layer displacement due to underlying drusen. The red line demarcates the border of the largest drusen.

**Figure 3.**
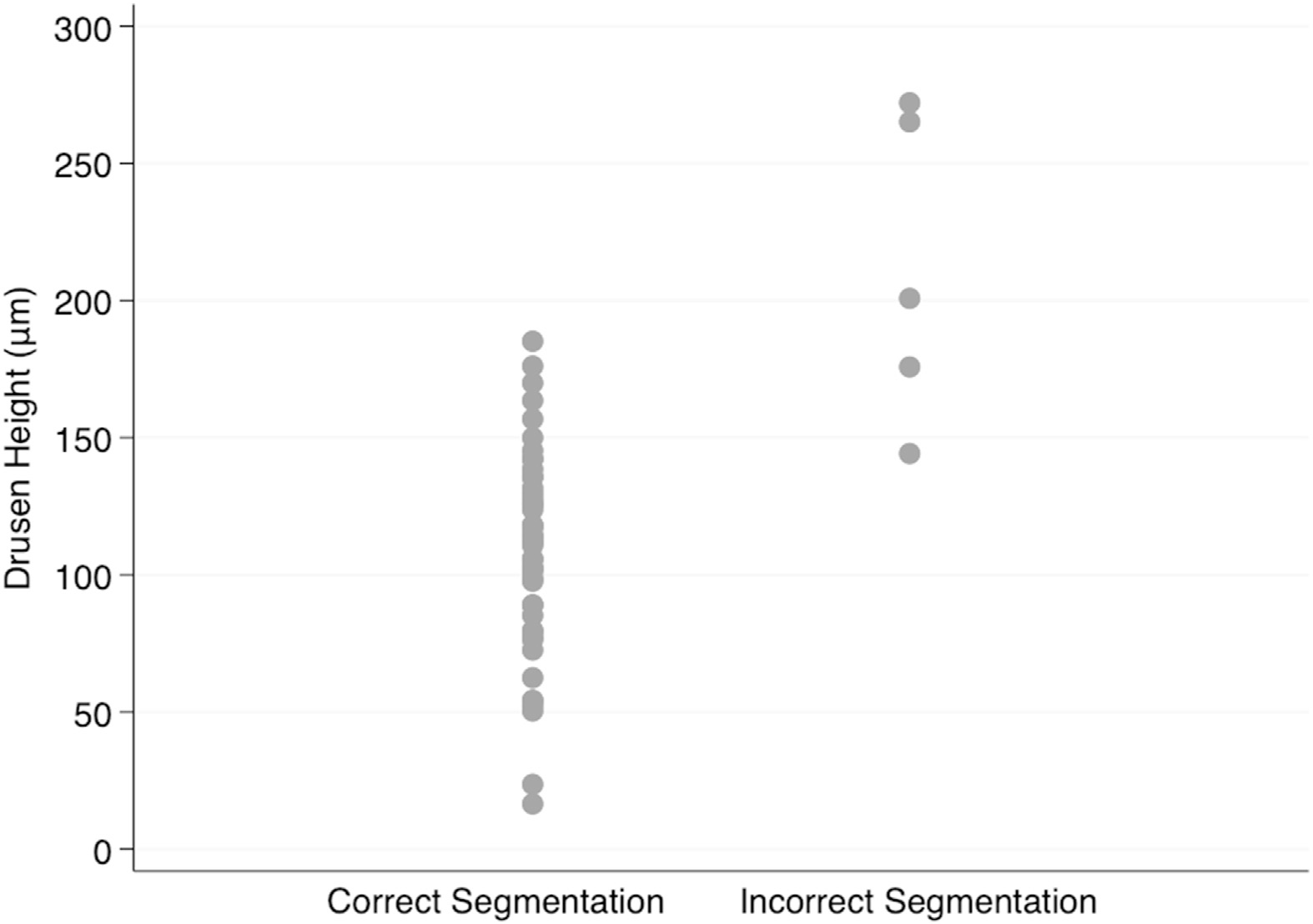
Distribution of the absolute drusen height on macular B-scan images according to the quality of segmentation.

Figure 1 demonstrates the distribution of absolute drusen height of the included images. The average height of drusen in this subset was 118.1 ± 49.5 μm. Figure 2 displays examples of changes on the inner macular layers caused by large drusen. Figure 3 shows the absolute drusen height on the selected macular B-scans according to the quality of segmentation (i.e., correct vs. incorrect segmentation). Five out of 53 images (9.4%) were incorrectly segmented by the Glaucoma Module Premium Edition algorithm. Images with drusen heights above 185 μm were all incorrectly segmented, and images with drusen heights below 145 μm were all correctly segmented. There was some overlap in the 145 to 185 μm range between the 2 groups, with 2 out of 9 B-scan images (22%) incorrectly segmented. There was a significant difference in the average height of the correctly segmented and incorrectly segmented groups (108.3 ± 5.4 μm vs. 203.0 ± 20.9 μm; *P* < 0.001).

**Figure 4.**
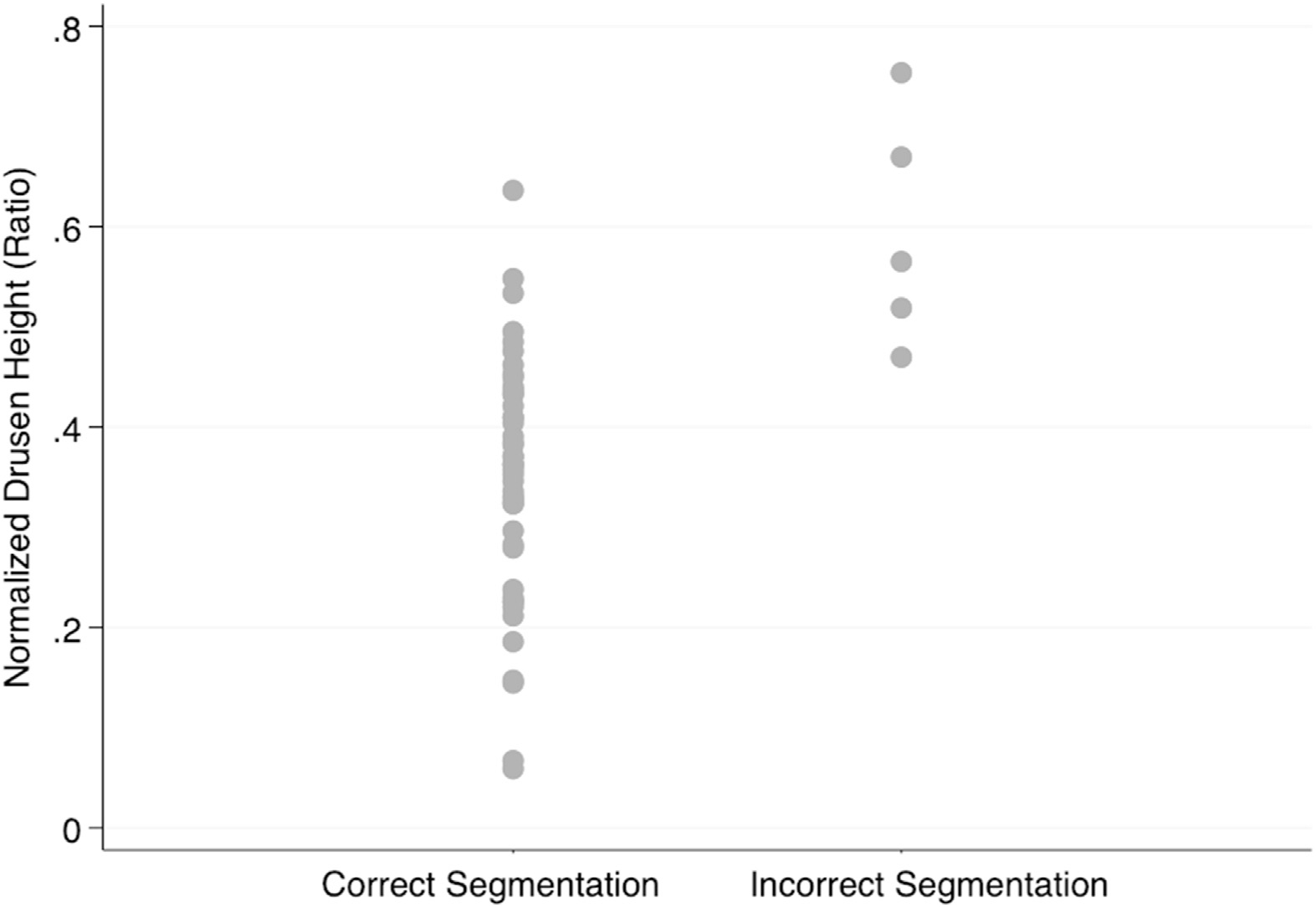
Distribution of the drusen height normalized by total retinal thickness on macular B-scan images according to the quality of segmentation.

Figure 4 plots the normalized drusen height according to the quality of segmentation; the normalized drusen height was estimated by dividing the absolute drusen height by the total retinal thickness at the location of the tallest drusen. The normalized drusen height in the correctly segmented group ranged from 0.06 to 0.64, whereas it ranged from 0.47 to 0.67 in the incorrectly segmented group. Overall, the ratio of drusen height to total retinal thickness was higher in the incorrectly segmented group. There was considerable overlap between the 2 groups in the 0.45 to 0.65 range, with 4 out of 13 images (31%) incorrectly segmented. The normalized drusen height was significantly different between the correctly and incorrectly segmented groups (0.35 ± 0.017 vs. 0.57 ± 0.037; *P* < 0.001).

**Figure 5.**
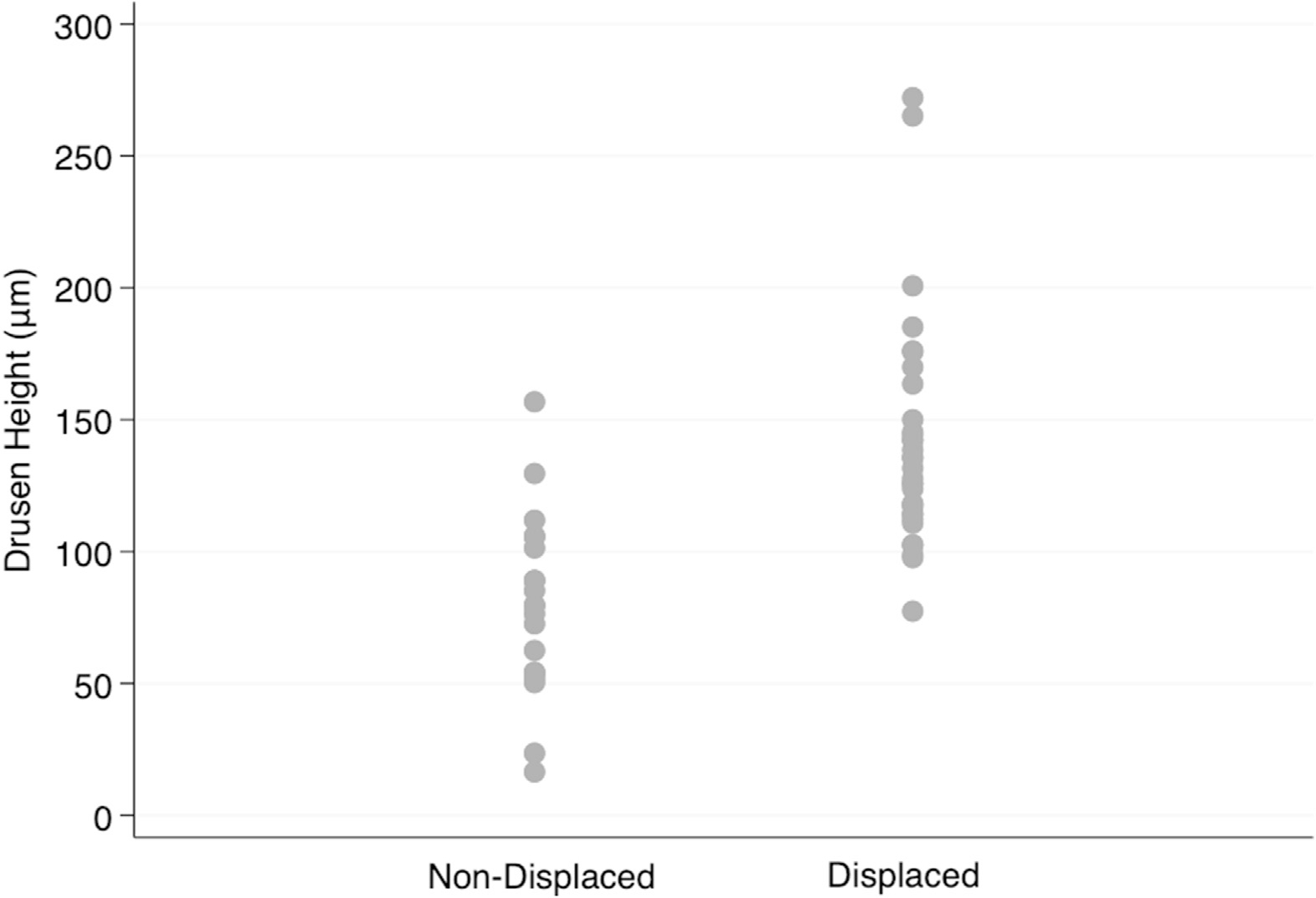
Distribution of the absolute drusen height on macular B-scan images according to presence or lack of tissue displacement.

**Figure 6.**
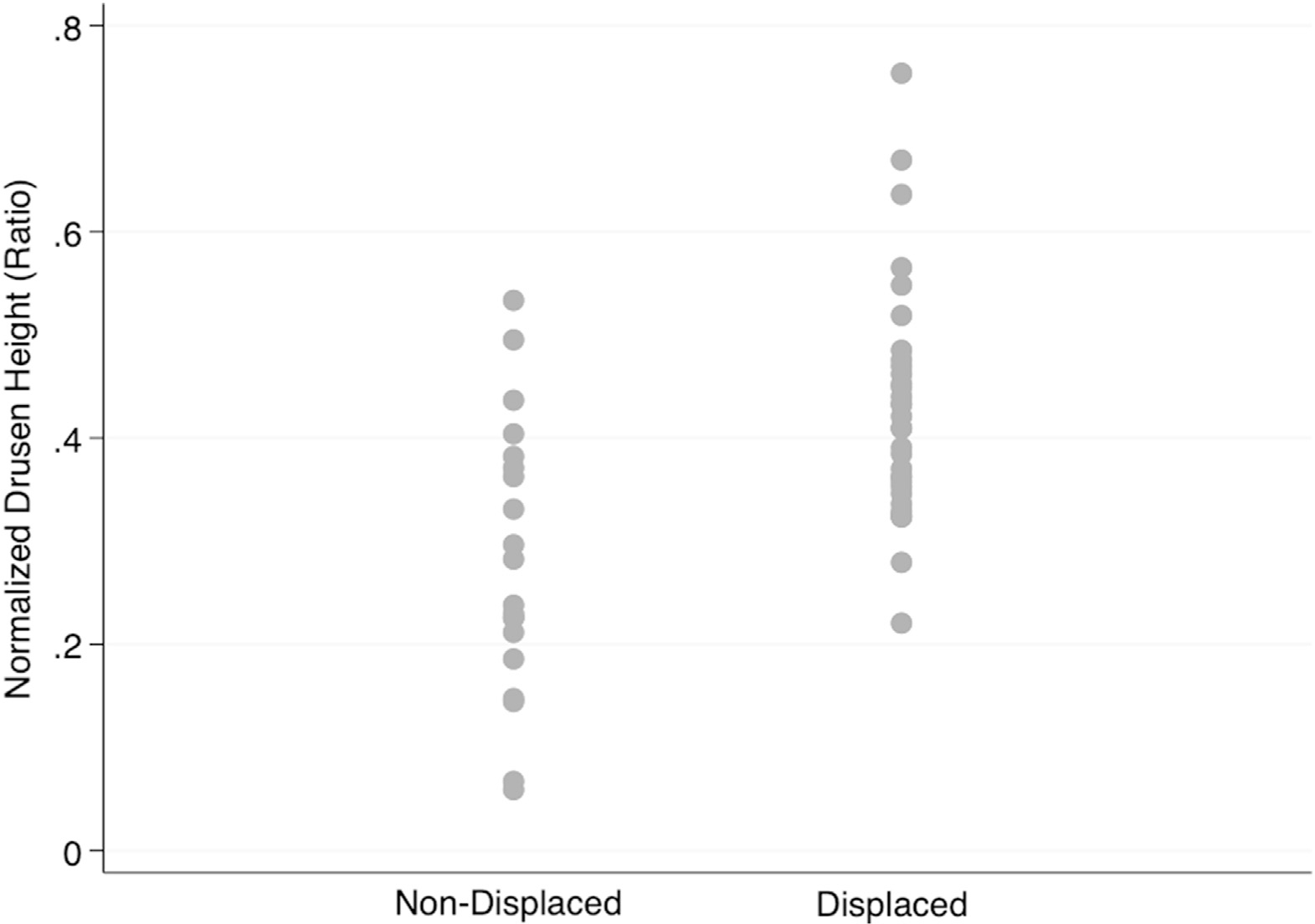
Distribution of drusen height normalized by total retinal thickness on macular B-scan images according to presence or lack of tissue displacement.

Figures 5 and 6 demonstrate the distribution of drusen height on macular B-scans categorized based on whether there was a visible displacement of the inner retinal layers. While Figure 5 shows the absolute drusen height on the y-axis, Figure 6 provides normalized drusen height (drusen height divided by total retinal thickness ratio). The range of absolute drusen height varied from 16 to 176 μm and the normalized drusen heights ranged from 0.06 to 0.50 on B-scans with nondisplaced inner retinal layers. On the other hand, on B-scans with displaced inner retinal layers, absolute drusen height varied from 77 to 272 μm, and normalized drusen height ranged between 0.22 and 0.75. There was considerable overlap between the nondisplaced and displaced groups in the 77 to 176 μm range (0.22 to 0.50 for normalized value). No tissue displacement was observed below a drusen height of 77.4 μm (normalized drusen height, 0.22). The difference between the nondisplaced and displaced groups in both absolute (84.4 ± 8.6 vs. 139.3 ± 7.7 μm; *P* < 0.001) and normalized scales (0.29 ± 0.029 vs. 0.42 ± 0.020; *P* < 0.001) was significant. Table 2 summarizes the frequency of poor segmentation and tissue displacement as a function of drusen height.

### Effect of Outer Retinal Atrophy on Inner Retinal Layers

**Figure 7.**
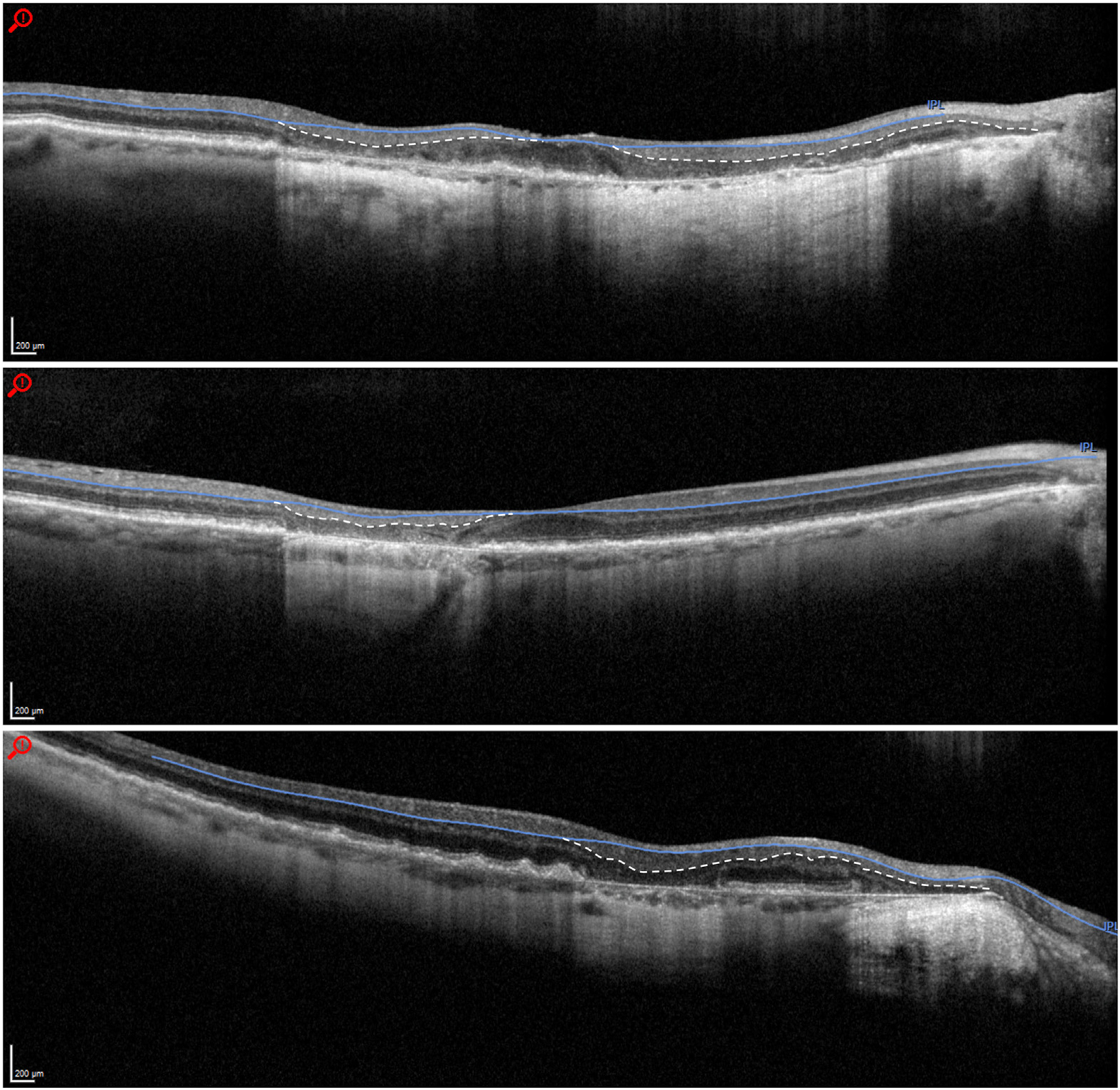
Examples of incorrect segmentation due to geographic atrophy causing posterior prolapse of the inner retinal layers. The white dashed lines show where the correct inner plexiform layer/inner nuclear layer boundaries are.

Geographic atrophy was present in 31 images, resulting in thinning and collapse of the outer retinal layers with subsequent outward prolapse of the inner retinal layers. There was distortion of the inner retinal architecture on all the B-scans. In 27 out of 31 B-scans (87.1%), the segmentation line of the IPL/INL boundary did not follow the prolapsed and more posteriorly displaced inner nuclear/outer plexiform boundary, thus resulting in incorrect segmentation. Figure 7 shows some examples of these segmentation errors in eyes with outer retinal atrophy.

## Discussion

We investigated the effect of nonexudative AMD on the inner retinal structures and IPL/INL boundary segmentation. This is an inherently challenging task, and we limited our study to eyes with dry AMD to be able to better describe the changes of interest in the inner macula. We also limited our study to explore the changes in the IPL/INL boundary because our preliminary analyses demonstrated that if this boundary was not affected in any way, it was very unlikely for the other inner retinal layer boundaries to be affected. Our main finding is that large drusen or geographic atrophy may result in distortion of the overlying inner retinal layers and lead to incorrect segmentation of the IPL/INL boundary. This inaccurate segmentation could potentially result in erroneous conclusions when macular OCT imaging is used in the detection of glaucoma or its progression as glaucoma deterioration manifests itself mainly within the inner retinal layers.^2,3,6,16,17^

The fact that outer retinal diseases such as AMD can artifactually alter the architecture of the inner retina or cause inaccurate automated segmentation of the inner retinal layers is known. However, this topic has not been previously investigated formally. Nonexudative AMD can manifest as drusen or outer retinal atrophy on OCT images, both of which can alter the analysis of the inner retina in different ways. We found that most small and intermediate drusen did not affect the inner retinal topography. On the other hand, larger drusen resulted in displacement or compression of the inner retinal layers. These alterations caused incorrect automated segmentation of the IPL/INL boundary in some eyes. Based on our study sample, automated segmentation has about a 20% chance of failure if the drusen height is 145 to 185 μm and is likely to fail with drusen heights above 185 μm. Because the influence of drusen on the inner retinal layers can depend on the total thickness of the retina in the area of the drusen, we also normalized drusen height by total retinal thickness in the region of drusen to account for this confounding issue. When drusen height was normalized by total retinal thickness, segmentation was likely to fail when the drusen to total retinal thickness ratio was 0.45 or above. There was a significant difference between the correctly segmented and incorrectly segmented groups in the average absolute height (108.3 ± 5.4 μm vs. 203.0 ± 20.9 μm; *P* < 0.001) as well as the normalized drusen height (0.35 ± 0.017 vs. 0.57 ± 0.037; *P* < 0.001).

This study found that segmentation was accurate in the presence of small to intermediate drusen and only failed in the presence of large drusen heights. A study by Alten et al^18^ reporting on segmentation errors of the IPL on OCT angiography images found a significant difference in drusen height between correctly and incorrectly segmented images. However, in their study, there were 26 images that were inaccurately segmented with an average drusen height of 98.1 ± 3.7 μm. Their rate of inaccurate segmentation was much higher and failed at a lower average drusen height compared with our study. Likewise, another study by Lauermann et al^19^ found that 58% of images with early or intermediate AMD had IPL segmentation error on OCT angiography. The differences in the average drusen height leading to inconsistencies in tissue boundary segmentation could be due to differences in the software used for segmentation in the current vs. the 2 aforementioned studies. Both of the above studies used the AngioVue software on the RTVue XR Avanti spectral domain OCT device (Optovue).

At times, we found that even if IPL/INL boundary segmentation were accurate, large drusen could still cause compression and mild displacement of the inner retinal layers. A significant proportion of the images in our study sample were found to have displaced inner retinal layers. Images were likely to show displacement with drusen heights above 176 μm and a normalized drusen height ratio of 0.5 or higher. For example, within the drusen height range of 77 to 176 μm, 75% of the B-scans showed varying degrees of displacement of the IPL/INL boundary. Using normalized drusen height, 65% of the B-scans with a normalized ratio of 0.22 to 0.50 displayed some displacement of the IPL/INL boundary. This might lead to decreased estimated thickness of the GCC due to tissue compression rather than from glaucomatous damage. Lee et al^20^ found that patients with dry AMD had significantly thinner GCIPL thickness compared with controls, with a mean reduction of 10%, and that larger drusen area and volume significantly correlated with thinner GCIPL thickness. These changes were not associated with structural alterations in the optic disc or glaucomatous changes in visual field parameters, although visual field defects such as foveal or parafoveal scotomas were noted. The decreased thickness in these patients may be interpreted by clinicians as progressive macular thinning due to advancing glaucoma severity rather than resulting from AMD and may thus affect the management of these patients in a negative way.

Another feature of AMD, particularly in advanced cases, is geographic atrophy. Atrophy causes the outer retinal layers to thin out and collapse, which in turn causes the inner retinal layers to prolapse outward. Images with atrophy had a much higher rate of incorrect segmentation than drusen with 87% of images displaying incorrect segmentation. This is consistent with the study by Lauermann et al^19^ that examined segmentation accuracy in various outer retinal diseases and found that geographic atrophy had the highest rates of segmentation error of the IPL (100%; n = 6), although this study was carried out on OCT angiography images. In our study, the automated segmentation line in images with atrophy typically did not follow the prolapsed contour of the IPL/INL boundary, but rather followed the projected course of the boundary as if the retinal architecture were normal. This resulted in an inwardly misplaced IPL/INL boundary. The calculated inner retinal layer thickness measurements are therefore falsely decreased.

Software programs with automated retinal layer segmentation need to be able to recognize the confounding effects of outer retinal disease. Ideally, these programs could then alert the clinician to images that are at risk of these confounding effects, similar to the alerts of low test reliability on visual field examinations.^21^ With further development of deep learning algorithms, it would be expected that this task could become automated in an efficient way. Analysis of the magnitude and prevalence of these confounding effects is a key first step in improving the interpretation of macular OCT parameters in eyes with outer retinal disease.

One of the limitations of our study includes the specific focus on nonexudative AMD. Future investigations will need to explore the effects of other retinal pathologies on inner macular thickness measurements as well as the interactions between multiple retinal diseases. Additionally, this study focused on drusen height as the main pathological effect. Drusen volume may need to be investigated as another predictor affecting inner retinal analysis. Also, there is no accessible and easy way yet to quantify the vertical extent of outer retinal atrophy due to nonexudative AMD, and hence, we were not able to quantify the inner retinal changes as a function of AMD severity. Lastly, all the analyses in this study were performed on the macular volume scans from Heidelberg Spectralis OCT using the built-in Glaucoma Module Premium Edition software for segmentation; therefore, the results of this study may not be generalizable to other imaging platforms.

Inner retinal layer thickness measurements on macular OCT volume scans play an increasingly important role in the evaluation of glaucoma severity. Outer retinal diseases, such as AMD, can alter the retinal topography and affect the accuracy of segmentation of the inner retinal layers, which can in turn influence the interpretation of such data by clinicians. Large drusen may cause segmentation errors and compression of the inner macular layers, leading to decreased thickness measurements that may be misinterpreted as atrophic changes of the inner retina. However, segmentation of inner retinal layers remains accurate in eyes demonstrating drusen with small to medium heights. Geographic atrophy causes incorrect automated segmentation in a high proportion of eyes with extensive outer retinal atrophy. Clinicians should be cognizant of the effects of outer retinal disease on the inner retinal layers and consider our findings when interpreting the results of automated macular segmentation in eyes with outer retinal diseases such as nonexudative AMD.

## Supplementary Material

Supplementary Figure 1

## Figures and Tables

**Table 1.** Demographic and Clinical Features of the Patients Enrolled in This Study*

Demographic measure	N (%)

Age, yrs	
< 60	2 (3.1%)
60–69	7 (10.8%)
70–79	26 (40.0%)
80–89	23 (35.4%)
90+	7 (10.8%)
Sex	
Male	31 (47.7%)
Female	34 (52.3%)
Ethnicity	
White	52 (80.0%)
Asian	3 (4.6%)
Hispanic	1 (1.5%)
Unspecified/Other	9 (13.8%)
AMD stage	
Early	10 (12.2%)
Intermediate	43 (52.4%)
Advanced atrophic without subfoveal involvement	19 (23.2%)
Advanced atrophic with subfoveal involvement	10 (12.2%)

AMD – age-related macular degeneration.

* Percentages for age, sex, and ethnicity were calculated out of 65 patients. The percentages for AMD stage were calculated out of 82 eyes.

**Table 2.** Number of B-scan Images With Correct Segmentation, Incorrect Segmentation, Nondisplaced Inner Retinal Boundary (Inner Plexiform/Inner Nuclear Junction), and Displaced Inner Retina as a Function of Absolute or Normalized Drusen Height

	Correct segmentation	Incorrect segmentation	Nondisplaced inner retina	Displaced inner retina

Absolute drusen height (μm)				
0–50	2	0	2	0
50–100	15	0	12	4
100–150	26	1	5	21
150–200	5	1	2	4
200–250	0	2	0	1
> 250	0	1	0	2
Normalized drusen height*				
0–0.15	4	0	4	0
0.15–0.30	10	0	8	3
0.30–0.45	26	0	6	19
0.45–0.60	7	3	3	7
> 0.60	1	2	0	3

* Drusen height values were normalized by dividing the drusen height by the total retinal thickness at the location of the drusen, as defined by the shortest perpendicular distance from the outer retinal pigment epithelium–Bruch’s membrane complex to the inner limiting membrane.

## Abbreviations and Acronyms:

AMD: age-related macular degeneration
BM: Bruch’s membrane
GCC: ganglion cell complex
GCIPL: ganglion cell–inner plexiform layer
ICD: International Classification of Diseases
INL: inner nuclear layer
IPL: inner plexiform layer
OCT: Optical coherence tomography
RNFL: retinal nerve fiber layer
RPE: retinal pigment epithelium
