# Supplementary figures and images for: Measurement of the Inner Macular Layers for Monitoring of Glaucoma: Confounding Effects of Age-Related Macular Degeneration
Source: Ophthalmol Glaucoma. Author manuscript; Available in PMC 2023 Feb 17. (PMC9937646; doi:10.1016/j.ogla.2022.06.006)

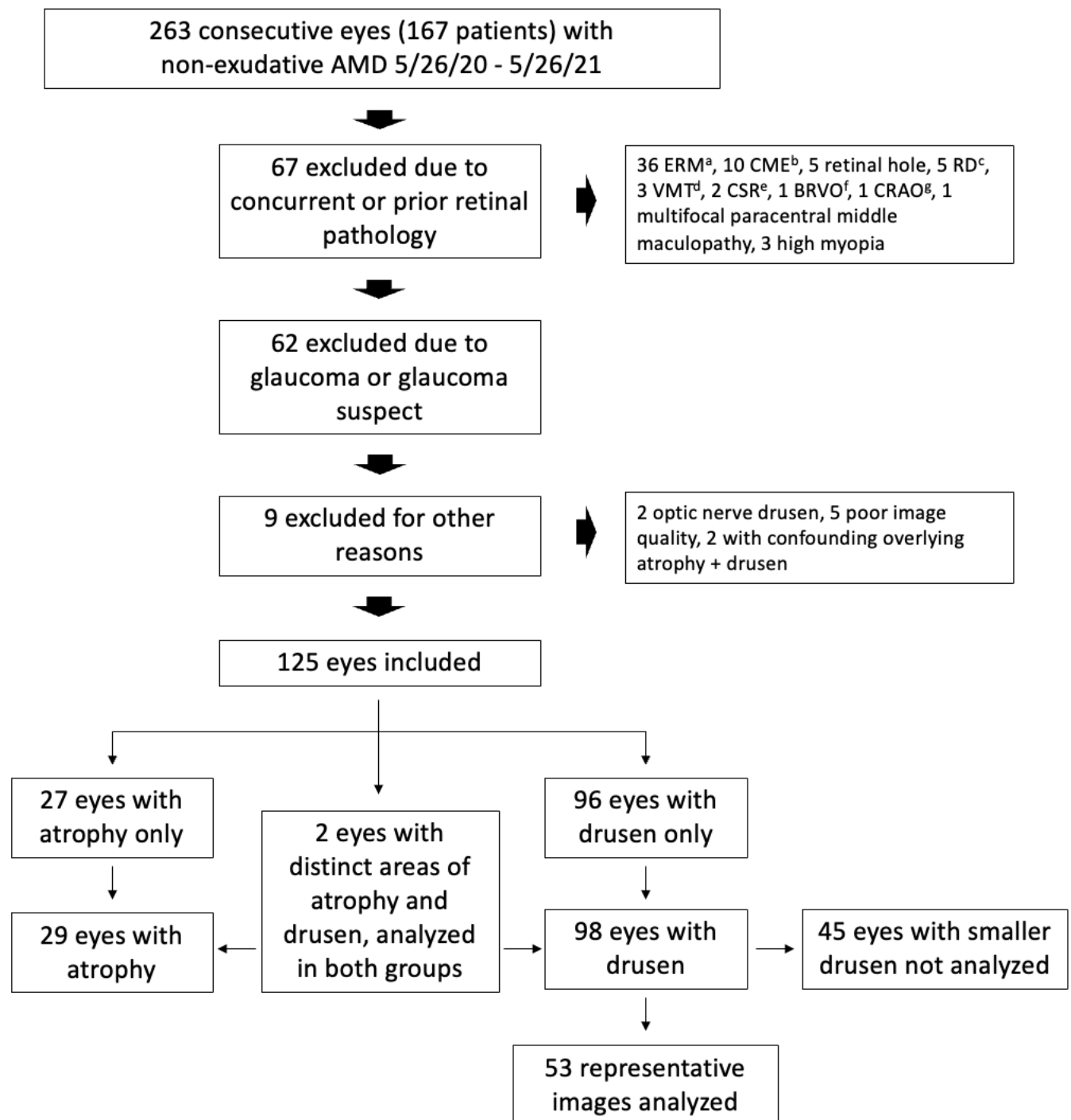

Supplement: Supplementary Figure 1 [file NIHMS1863561-supplement-Supplementary_Figure_1.pdf]
